# Inquiry into Prof. Herrick’s Theory of Cholera

**Published:** 1849-09

**Authors:** Henry Ritchie

**Affiliations:** Chicago, Illinois


### ARTICLE IV.

*An inquiry into Professor Herrick's theory of Cholera, in connection with Ozone.* By HENRY RITCHIE, M. D., of Chicago, Illinois.

The Professor remarks, (after a detailed account of ozone, relative to its discovery, and its agency in producing epidemic influenza, and the inference that it probably might be the cause of cholera:) "Having assumed as, we do, that ozone is the cause of cholera, we shall now, after refering briefly to a few physiological facts, attempt to explain its mode of action."

The Professor then gives us the physiological changes that ensue in the capillaries, by which the arterial is converted into venous blood. And further remarks, "It is evident that these important changes would not occur, nor could the functions of any organ be properly performed, without a due supply of arterial blood constitued of its normal constituents and furnished by the lungs with the proper amount of uncombined oxygen."

He moreover says, "It is absurd to suppose, for instance, that in this disease (cholera) as in health, oxygen and the constituents of the blood and tissues combine chemically in the capillaries, since no animal heat is generated in these vessels, as the result of such combination. Yet it is evident from the appearance of the blood in the disease that such changes do occur, and that arterial is converted into venous blood more effectually and rapidly, even, than in health." To prove the position that heat is not generated in the capillaries, he cites the coldness of the breath, and surface of the body, and then follow these remarks :—

"Yet both sides of the heart, and all the blood vessels, arteries as well as veins, are found to contain, both before and after death, dark venous blood. These facts, taken in connection with numerous others, show as conclusively as the nature of the case will admit, that these changes in the blood take place in the arteries and left side of the heart during its passage to, and before it reaches the systemic capillaries. It is evident that in health there exists in the vessels a force by the action of which chemical union is effected between elements, which, though in contact and intimately blended together, do not combine in passing through the heart and large arterial trunks, in which no such force exists.

Now if it can be made to appear in some diseases, as in cholera, a force equal to, or stronger than this, acts upon the arterial blood during its passage to, and before it escapes the systemic capillaries, causing a chemical combination of its constituents with oxygen, there is no difficulty in explaining how ozone, one of the most powerful oxydizing agents known, produces cholera.

When present in the air, it passes into, and is mixed with the arterial blood like pure oxygen, and being, as it is, a compound of oxygen and hydrogen, easily decomposes; slight causes effect a separation of its elements, and the consequent production in the arteries of new compounds of oxygen and the constituents of the blood, similar to those produced naturally in the capillaries.

During the decomposition of the ozone, its oxygen being in the nascent state and consequently more ready to form new combinations, changes the arterial blood to venous, during its passage along the heart and arteries, previous to reaching the systemic capillaries."

The Professor says that his pathology of cholera is sustained by the symptoms during life, and examinations after death. The burning sensation at the pit of the stomach shows that so far as their feelings and desires are indicative of their (the patients) condition, heat must be generated in the heart and large arteries of the chest and abdomen as the result of chemical changes, whilst the entire absence of it, on the surface, at the extremities, and in the mucous membrane lining the cavities, are conclusive evidences that no such changes take place in the capillary vessels.

Now if this is not jumping to conclusions with a vengeance, then we are no judge. Archimedes said that if he had a fulcrum for his lever he could heave the globe. Grant some gentlemen their premises and, in argument, they can almost perform a parallel feat.

The Professor assumes, in the first place, that in diseases, particularly in that of cholera, there is a force existing somewhere, either in the left heart or large arteries, that subverts the office of the capillaries; that the blood by this capricious force is changed into venous before it reaches the capillaries; and, that in consequence of this conversion of the arterial into venous blood, heat is evolved somewhere in the neighborhood of the heart or large arteries, for patients complain of this heat.

He assumes that ozone is this force: from its being a compound of oxygen and hydrogen, it is easily decomposed, slight causes effecting it, and new compounds of oxygen and the constituents of the blood arise, converting it into venous blood. According to the Professor's theory, the whole pathology of cholera is the conversion of arterial into venous blood in a part of the system where this is not want to happen. All the phenomena of this disease are owing to this vicarious action induced by ozone, the vomiting, cramping, purging, etc. But the intellect throughout the attack is perfectly normal, notwithstanding nothing but venous blood is flowing through the vessels of the brain: is not this a little strange?

How does the Professor's theory comport with truth? The dark color of the venous blood in the normal state of the system, is supposed to be owing to the excess of carbonic acid gas contained in it; and its conversion into arterial in the capillaries of the lungs is owing to its parting with its excess of carbonic acid and the replacement of oxygen gas. It is an admitted fact that in the capillaries, the laws that govern nutrition, secretion and excretion are directly applied; and carbon being one of the results of disintegration or decomposition of the tissues, and also an effete substance to be carried out of the system as deleterious, two organs are supplied for this purpose, viz., the lungs and liver. Carbon unites with two portions of oxygen, conveyed to this system of vessels by the arteries, forming by their union carbonic acid gas, which combination generates animal heat.

Then the great difference between arterial and venous blood is this, that in the former there is an excess of oxygen; whilst in the latter a deficiency of oxygen and excess of carbonic acid which the venous blood is loaded with as an effete production to be eliminated from the system.

It is a well established fact, that in the lungs the venous blood gives off carbonic acid, the union of carbon and oxygen having taken place in the systemic capillaries, and not, as was formerly supposed, in the lungs.

If the ozone theory is true, we should like the professor to account rationally, and not by assumption how the whole volume of the blood in cholera patients is changed into dark venous blood. Ozone according to his own showing, is a tritoxide of hydrogen, that is three atoms of oxygen united with one of hydrogen; this he says, is inhaled into the lungs and passes into the circulation, and as a matter of course, at the same time the usual amount of uncombined oxygen is also taken into the lungs and absorbed, as constitutes the normal function of this organ.

Here then we have by the inhalation of ozone also diffused in the atmosphere, three atoms more of oxygen than is usually absorbed into the blood to change it from venous to arterial. And yet this great excess of oxygen, strange to say, converts the whole blood into venous, according to the professor, though his theory in the meantime maintains that nutrition, secretion, and excretion are all in abeyance to the disease: carbon cannot therefore be furnished to form one of the necessary elements for carbonic acid gas.

Now, notwithstanding the professor's theory, if experiments teach us any thing, and are of any value, the whole mass of blood instead of being dark crimson or black, should be on the contrary, a vivid scarlet, for this is always the result of the inhalation of oxygen gas, and some physicians on these very grounds have suggested as a curative agent the inhalation of oxygen gas.

The professor states as an evidence of the generation of heat in the large arteries, the burning sensation in the pit of the stomach, will the professor point out the large arteries from which this comes. Why do not patients complain of heat about the heart? we never heard of any complaining of that, but they do complain of great heat about the bowels: will the professor point out the large arteries from which this comes.

The professor says that there is absence of heat in the mucous membranes lining the cavities, then why do the patients crave as he says they do water, if the cavities lined with mucous membranes are so cold: we should rather conclude that it was to allay the sensation of heat in the lining membrane of the stomach and bowels.

The professor says that there is some particular force (he does not say what this force is, however,) in the capillaries by which arterial is converted into venous blood. Now he says if it can be made to *appear* (aye, there is the rub) that in some diseases as in cholera, for instance, a force equal or stronger than this acts upon the arterial blood during its passage towards the capillaries, causing a chemical combination of its constituents with oxygen, *why then* there is no difficulty in explaining how ozone produces cholera. Now the professor goes on to show this force, which he has established to be ozone without a doubt, and how it steps in and supersedes the vital action of the capillaries.

How does the professor prove this? why in this way; that by its being granted that ozone is one of the most powerful oxydizing agents known, and by its being granted that it passes in and is mixed with the arterial blood, and as it is a compound of oxygen and hydrogen therefore it is decomposed; slight causes effecting a separation of its elements, and union takes place with some of the constituents of the blood, carbonizing the whole mass. Therefore, as a sequence to the above (which is very conclusive) the arterial blood in the heart and large arteries is converted into venous blood, and cholera ensues.

Does the professor call this logic? we can prove just as logically that carbonic acid in the atmosphere produces cholera, or sulphureted hydrogen, or any other gas. Let us try carbonic acid gas, this gas is a deutoxide of carbon: when it prevails in the atmosphere, it is inhaled into the lungs (if it is not in too large quantities, for then it causes spasm of the glottis.) Now this being a force stronger than the force in the capillaries, it acts upon the arterial blood during its passage to, and before it reaches the systemic capillaries, causing a chemical combination of its constituents with oxygen. There is then no difficulty in explaining how carbonic acid, one of the most powerful agents for turning arterial blood venous, produces cholera.

When present in the air, it passes into and is mixed with the arterial blood like pure oxygen, and being as it is a compound of oxygen and carbon easily decomposes: slight causes effect a separation of its elements, and the consequent production in the arteries of new compounds of oxygen and the constituents of the blood similar to those produced naturally in the capillaries; during the decomposition of the carbonic acid its oxygen being in the nascent state, and consequently more ready to form new combinations, changes the arterial blood to venous during its passage along the heart and arteries &c., &c. Now we do flatter ourselves that this is a more plausible argument than the professor's, for it has some facts that would appear to substantiate it; for instance the known fact of carbonic acid changing the color of arterial blood, whereas the professor's theory is purely ideal without a single fact to substantiate it. The professor does not deny that cholera is preceded generally by diarrhœa, and if these premonitories can be checked, the threatened attack passes off: will the professor explain this according to his theory. Will the professor give us the *rationale* of the action of sulphur in neutralizing not the agent ozone, but the effect that this agent produces on the system. A gun-shot wound is received does the professor recommend that we should apply our remedies to the bullet, or to the effects of the bullet? as well might we treat the bullet to cure the gun-shot wound, as to give an antidote for the effects of ozone on the system.

If it could be shown that ozone was the cause of cholera, and that sulphur would decompose this agent: then a sensible use might be made of it, or at least a plausible one merely as a prophylactic and not as a curative.

It will not do to establish practice upon theory, for men will run into all kinds of absurdities; as an example *similia similibus curantur*, here a most absurd system of practice is built upon the purest hypothesis, but let it be built upon facts and then it will be as firm as the house founded upon the rock, and the gusts of empiricism that sweep the land will be hurled from its solid foundation as the spray from the beetling ocean crag.

